# Special Considerations for Advanced Heart Failure Surgeries: Durable Left Ventricular Devices and Heart Transplantation

**DOI:** 10.3390/jcdd11040119

**Published:** 2024-04-15

**Authors:** Armaan F. Akbar, Alice L. Zhou, Annie Wang, Amy S. N. Feng, Alexandra A. Rizaldi, Jessica M. Ruck, Ahmet Kilic

**Affiliations:** Division of Cardiac Surgery, Department of Surgery, The Johns Hopkins Hospital, 1800 Orleans Street, Zayed 7107, Baltimore, MD 21287, USA; aakbar3@jhmi.edu (A.F.A.); azhou18@jhmi.edu (A.L.Z.); awang118@jhmi.edu (A.W.); sfeng26@jhmi.edu (A.S.N.F.); arizald1@jh.edu (A.A.R.); jessicaruck@jhmi.edu (J.M.R.)

**Keywords:** left ventricular assist device, heart transplantation, risk factors, right ventricular failure, valvular disease, small left ventricular cavity, temporary mechanical circulatory support

## Abstract

Heart transplantation and durable left ventricular assist devices (LVADs) represent two definitive therapies for end-stage heart failure in the modern era. Despite technological advances, both treatment modalities continue to experience unique risks that impact surgical and perioperative decision-making. Here, we review special populations and factors that impact risk in LVAD and heart transplant surgery and examine critical decisions in the management of these patients. As both heart transplantation and the use of durable LVADs as destination therapy continue to increase, these considerations will be of increasing relevance in managing advanced heart failure and improving outcomes.

## 1. Introduction

Modern definitive therapies for advanced heart failure include heart transplantation and durable mechanical circulatory support (MCS). Heart transplant surgery is technically challenging, and there are numerous surgical, device, donor, and recipient-level factors that impact operative decision-making and perioperative care.

While heart transplantation remains the gold standard advanced heart failure surgery, limited donor heart availability and technological advancements have allowed for the emergence of durable MCS use [1]. Durable left ventricular assist devices (LVADs) provide long-term hemodynamic support and have been utilized in heart failure patients as a bridge to transplantation and destination therapy and as a bridge to further decision-making [2]. LVAD implant surgery is complicated by patient anatomy and comorbid conditions, and surgeons and care teams must thoroughly assess appropriate candidates and predict potential complications that may occur during the operation and in the perioperative period. This review discusses special risk factors and considerations for durable LVAD and heart transplant surgery.

## 2. Considerations for Left Ventricular Assist Device Implantation

### 2.1. Right Ventricular Failure

Right ventricular failure (RVF), a major complication of LVAD placement, is thought to affect up to 40% of LVAD-supported patients and is associated with poor outcomes, including reduced LVAD function, impaired organ perfusion, and a 6-month mortality of up to 29% [3,4,5,6]. As a result of LVAD function, increased flow in the systemic circulation can lead to right ventricular (RV) volume overload, and excessive left ventricular (LV) drainage can shift the interventricular septum into the LV, leading to impairments in RV systolic/diastolic function. To maximize benefit from LVAD support, it is essential to identify patients at risk for development of RVF after implant. Existing risk prediction models incorporate variables based on hemodynamics, echocardiography, biochemistry, and clinical assessment. Some variables found to have an association with RVF include preoperative circulatory support use, prior cardiac surgery, non-ischemic heart failure, female gender, central venous pressure/pulmonary capillary wedge pressure > 0.63, blood urea nitrogen > 39 mg/dL, pre-implant tricuspid regurgitation (TR), and RV geometry [4,6,7,8,9]. Pulmonary artery pulsatility index (PAPi), a relatively novel hemodynamic index defined as the ratio of pulmonary artery pulse pressure (pulmonary artery systolic pressure [PASP]—pulmonary artery diastolic pressure [PADP]) to right atrial pressure (RAP), has also been reported to be independently associated with RVF after LVAD implant. The equation for PAPi is (PASP−PADP)/RAP. In a cohort of continuous-flow LVAD recipients, Kang et al. showed that PAPi higher than 3.4 was associated with a lower risk of RVF, and PAPi was more predictive in patients receiving inotropic support [10]. Right ventricular stroke work index (RVSWI) is another metric that reflects RV contractility that is derived from right heart catheterization (RHC) parameters. Several studies have shown that RVSWI is a predictor of mortality and RVF following continuous-flow LVAD implantation [7,11]. Despite the abundance of identified risk factors and prediction models for RVF development in LVAD patients, models yield differing results; a major issue is that RVF is heterogeneously defined and there is a need for a standardized definition to better select patients for LVAD and identify those who may decompensate [12]. Several major risk prediction models are summarized in Table 1.

In patients with RVF unresponsive to medical therapy, in the post-op period, consideration must be made for temporary right ventricular support. Options include veno-arterial extracorporeal membrane oxygenation (VA-ECMO), temporary right ventricular assist device (VAD) with single or dual-lumen cannula, Impella CP, RP and 5.0 (Abiomed, Danvers, MA, USA), TandemHeart (CardiacAssist, Pittsburgh, PA, USA), and Protek Duo (CardiacAssist, Pittsburgh, PA, USA) [13]. Potential complications and invasiveness of the implant technique of each device must be weighed with the patient’s hemodynamic needs and overall stability. Close follow up of LVAD patients is essential to detect RVF development early. Bhama et al. found a significantly better overall survival at 3 months in those implanted with an immediate right ventricular assist device compared to those who received delayed support (79% vs. 46%) [14], suggesting that the timing of support for RVF is of critical importance.

### 2.2. History of Stroke and Hypercoagulability

Like most implanted devices, LVADs activate the coagulation cascade, and pump thrombosis is a major complication of LVAD therapy. Pump thrombosis has been observed early [15] and late [16] after LVAD implantation and presents with hemolysis due to nonlaminar blood flow, increased pump power, and, in some cases, recurrent heart failure [17]. Pump thrombosis may additionally lead to thromboembolism and subsequent stroke. While clotting disorders and hypercoagulability are not contraindications for LVAD implant, they may plausibly increase the risk for thrombotic complications in the postoperative period. Few single-center studies have investigated the outcomes of LVAD implantation in patients with a history of hypercoagulability. Of those, results are mixed, with one reporting similar outcomes between hypercoagulable and non-hypercoagulable patients [18] and another finding higher deep-vein thrombosis and higher mortality in the hypercoagulable group [19]. Anticoagulation in these patients must be carefully managed, and further studies are necessary to determine the true risk of thrombotic complications in patients prone to developing clots.

Stroke is a leading cause of both death and disability worldwide [20], and due to the concomitance of coronary and carotid diseases, patients with a history of stroke often require cardiac surgery [21]. When considering LVAD implantation, the preoperative risk conferred by prior stroke is not fully understood [21]. In considering patients with prior stroke for LVAD placement, it is very important to quantify the severity of functional impairment. It is our recommendation that the timing of LVAD implant be delayed until maximal post-stroke recovery is achieved, such that recovery after LVAD implantation can be optimized. Though there is a lack of data on how prior stroke impacts outcomes after LVAD placement, it is unlikely that patients would benefit from LVAD therapy soon after a major stroke. This consideration must be balanced with the risk of mortality due to undertreated heart failure in these medically urgent patients. Perioperative ischemic and hemorrhage strokes are common complications of LVAD surgery and may be more likely and lead to even worse outcomes in patients with a history of prior stroke. Close monitoring and optimization of the functional status prior to surgery is necessary in this vulnerable population. Patients with prior ischemic stroke who are on anticoagulants, including warfarin and heparin, have increased perioperative considerations in regards to when to withhold and restart antithrombotic agents, balancing the necessity of thromboprophylaxis with increased bleeding risk [22,23]. In the postoperative period, effective anticoagulation in combination with subsequent stroke prevention and a close monitoring of functional status are crucial for patients with a history of stroke prior to implant [24].

### 2.3. Small Left Ventricular Cavity

Patients are considered to have a small left ventricular cavity when the measured left ventricular end-diastole dimension (LVEDD) is less than 5.5 cm. An LVEDD greater than 6 cm is considered to be more suitable for LVAD implantation because the ventricle has already generated thoracic adaptation, increasing cavity space to allow for LVAD implantation. LVEDD ≥ 6 cm also suggests that the etiology is likely to be dilated cardiomyopathy, which allows for uninterrupted flow of blood from the LV cavity into the LVAD [25]. Patients without thoracic expansion and a smaller chest area, who may have smaller LVEDD measurements, may require adjustments for anatomic fit and cannula position during the preoperative planning process. Approaches to surgery may include optimizing for a wider operating space by favoring a sternotomy over a thoracotomy or increasing space for LVAD implantation by entering through the pleural space [25]. The operating surgeon should adjust for pump inlet position and inlet angle when placing the LVAD to prevent flow obstruction, with the goal of orienting the inlet cannula towards the mitral valve [26,27,28]. Intraoperatively, transesophageal echocardiography monitoring until sternal closure ensures visualization of proper placement and reduces risk of RV stress [25]. Prophylactic pain management is suggested due to increased likelihood of rib and implant friction causing inflammation and postsurgical pain. As with all LVAD patients, long-term monitoring of suction events is recommended to assess for the progression of LV remodeling [27].

Various studies have found small LV to be a risk factor for 30-day morbidity and one-year mortality following LVAD implantation [29,30,31], with reduced LV volume potentially contributing to hemodynamically obstructed LVAD inflow, increasing flow turbulence and the development of pump thrombosis [32]. Computational modeling has shown that a small LV size is correlated with increased sheer stress histories of platelets and platelet residence time, suggesting potential thrombogenicity risk for patients with small LV [32]. Risk assessment of HeartMate 3 LVAD performance determined small LV size, defined as LVEDD less than 5.5 cm, as a significant factor contributing to one-year and two-year mortalities [33]. However, after accounting for a varying study design, smaller patient sample sizes, and conclusions drawn from computer simulations rather than in vivo models, other studies have found that LVEDD size does not increase mortality or morbidity with no difference in post-implant outcomes for patients with a smaller LV cavity [34]. Further studies are necessary to standardize the LVEDD size cutoff for evaluating small LV volume and distinguish between device-specific risks for stroke and pump thrombosis for patients with a small ventricle [35].

### 2.4. Preoperative Arrhythmia

Advanced heart failure patients selected for LVAD implantation often present with concomitant arrythmias. Atrial fibrillation (AF) is recorded in 21–54% of patients with advanced heart failure with reduced ejection fraction indicated for LVAD and is the most common sustained arrythmia both pre- and post-LVAD implantation [36]. Patients with long-term AF may experience improved left atrial function and negative remodeling due to the LVAD unloading the LV, subsequently reducing atrial pressure and dimensions while improving structural and electrical functions. Patients with advanced heart failure are also likely to have ventricular arrythmias prior to LVAD evaluation, specifically ventricular tachycardia (VT). Ventricular fibrosis has been linked to the initiation of ventricular tachyarrhythmias, in combination with increased filling pressures and wall stress, which LVAD therapy specifically serves to treat [37]. Additionally, 20–50% of patients are thought to experience new-onset or recurrent VT following LVAD implantation. Thus, preoperative considerations for VT patients include concomitant surgical ablation during LVAD placement and consultation with electrophysiology prior to LVAD implantation [38]. Additional preoperative concerns for patients with AF are related to anticoagulation usage and increased risk for postoperative complications, with pre-implant AF shown to be the strongest predictor of non-psychiatric postoperative delirium after cardiac surgery [39]. In patients with pre-implant AF, intraoperative maze and left atrial appendage clip during LVAD placement may be considered, although formal data on the efficacy of these interventions are lacking.

Prior AF history in patients with LVAD is shown to increase the risk of earlier heart failure hospitalization and mortality, but whether or not adverse event risk is independently caused by AF etiology rather than the LVAD procedure has not yet been elucidated [40]. While early studies suggested contractile dysfunction in the left atrial appendage may persist and pose an increased risk of thrombosis, in patients receiving recent LVAD pump designs with improved hemodynamic stability, a history of preoperative AF has not been shown to increase thromboembolism or major bleeding events [41,42]. Interestingly, a large-scale dataset of 18,378 patients, 41.7% of whom had preoperative AF, identified a lower in-hospital mortality in patients with AF with a decreased thromboembolic risk compared to patients without AF undergoing LVAD implantation [43]. A meta-analysis examining the effect of preoperative AF on outcomes after LVAD implantation found increased risk of gastrointestinal bleeding, but ultimately confirmed no significant association with post-implant mortality, stroke, or thromboembolic risk [44].

Prior history of VT has been shown to strongly predict postoperative VT, and affected patients selected for LVAD require monitoring for ventricular arrythmias, particularly in the initial 30-day postoperative period, with recurrent VT risk decreasing in the weeks and months of recovery following implantation [45]. However, despite the increased risk of VT recurrence, patients with a history of VT may not have increased risk of one-year overall mortality, necessitating further research to understand the relationship between VT and mortality [45]. Individual considerations for patients with sustained ventricular arrhythmias may follow the 2017 American Heart Association guidelines that provide a Class IIa advisory, suggesting implantable cardioverter-defibrillator therapy for LVAD patients is safe and beneficial [42]. However, ICD implantation in LVAD patients remains an area of active discussion and controversy. Areas for further exploration for LVAD patients with arrythmias include distinguishing risk factors for mortality and morbidity driven independently by arrythmia etiology as opposed to the hemodynamic changes introduced by LVAD therapy, while balancing complex advanced heart failure patient risk-profiles.

### 2.5. Pre-Existing Valvular Disease

The management of pre-existing aortic, mitral, and tricuspid valvular lesions in patients undergoing LVAD implantation is of particular importance, as they may influence the decision to proceed with LVAD support and may lead to concomitant valve repair or replacement.

Aortic insufficiency (AI) is a challenging problem in the utilization of continuous-flow LVADs. AI can be pre-existing or develop de novo due to the effects of the LVAD on heart physiology. Continuous-flow LVADs direct blood from the left ventricle into the aorta, creating a continuous transvalvular pressure gradient that is distinct from the pulsatile flow that the aortic valve experiences under normal physiology. This continuous pressure leads to fusion and deterioration of the aortic valve leaflets, ultimately resulting in regurgitant flow from the aorta to the LV [46]. Single-center studies have reported mild de novo AI development in 30–40% of patients in the months following both HeartMate II and HeartMate 3 implant, with 10–17% progressing to moderate–severe AI [31,47,48]. Echocardiography is essential in evaluating bi-ventricular diameters as well as pre-implant AI, as the severity may worsen after LVAD implantation. Many surgeons choose to correct moderate or worse AI at the time of LVAD insertion. This is typically done through concomitant bioprosthetic aortic valve replacement, though subsequent transcatheter aortic valve replacement in those with the development of AI after initial LVAD implant has been reported with success as well [49,50]. In all cases, achieving a range of pump speeds prior to discharge that allow for sufficient AV opening may prevent fusion of the valve leaflets and has been recommended as a way to prevent de novo AI development [46].

Functional mitral valve regurgitation (MR) is common in LVAD patients, with a prevalence of 36–54% reported among patients admitted with decompensated heart failure [51,52,53]. Heart failure leads to impaired ventricular remodeling, causing annular dilation and papillary muscle displacement, ultimately resulting in poor coaptation of the mitral valve and tethering of leaflets [54]. Mitral valve repair in functional MR is associated with a high recurrence of regurgitation and need for reoperation [55], and LVAD implantation unloads the left ventricle and reduces pulmonary artery pressure, leading to negative ventricular remodeling and reduction of LV volume. These changes serve to reduce the severity of MR. In the MOMENTUM 3 trial, which compared the centrifugal HeartMate 3 with the axial HeartMate II, 43.5% of patients had moderate or greater MR at implantation and did not undergo mitral intervention [56]. At 2 years of support, 9.4% of the HeartMate 3 group and 15.4% of the HeartMate II group had clinically significant MR. Given these results, concomitant valve repair is rarely required at the time of LVAD surgery, and patients should be monitored for persistence of MR in the months following implantation.

In advanced heart failure patients, the tricuspid valve is particularly vulnerable to insult. In the setting of right ventricular dilation, tricuspid annular dilation and incomplete coaptation may occur, leading to secondary regurgitation that may contribute to right heart failure [46]. Pre-existing TR could plausibly improve after LVAD implant, because LVADs serve to reduce LV end-diastolic pressure and pulmonary venous pressure, which decreases right ventricular afterload and can improve right ventricular function and contractility [57]. However, soon after LVAD surgery, flow is limited across the pulmonary vasculature and patients are volume-resuscitated, leading to RV dilation and exacerbation of TR. It has been reported that TR can acutely worsen following LVAD implantation, and persistent RV dilation has been observed at 30-days post-implant [58]. Right heart failure is associated with poor outcomes in LVAD patients, as discussed earlier, leading to the decision to perform concomitant tricuspid valve repair in select patients. Several single-center studies have found reduced improved postoperative outcomes and reduced early right heart failure in LVAD patients receiving concomitant tricuspid repair [59,60,61]. However, until definitive guidelines are set in place, there is no consensus on when to perform concomitant tricuspid repair. It is likely that patients receiving long-term LVAD support with moderate–severe TR would benefit from a concomitant tricuspid repair.

### 2.6. Temporary Mechanical Circulatory Support Bridging

Temporary MCS devices are part of the surgical armamentarium for the treatment of heart failure patients and are increasingly used as a bridge to durable LVAD therapy to improve survival [62,63]. These devices include IABPs, temporary ventricular assist devices (VADs), and veno-arterial extracorporeal membrane oxygenation (VA-ECMO) and are used as temporizing measures in heart failure patients prior to transplant or destination therapy with a durable VAD. Currently, there is no consensus regarding the choice of temporary MCS for the bridge-to-bridge (BTB) strategy. An overview of recent reports on short-term MCS (intra-aortic balloon pump, Protek Duo, TandemHeart, Impella CP, RP, 5.0, and extracorporeal membrane oxygenation [ECMO]) found that the bridge to durable LVAD occurred using all device groups but was more frequently performed in patients with end-stage cardiomyopathies than in patients with acute myocardial infarction or myocarditis [62]. The study found that a bridge to recovery or successful weaning was possible in at least one-quarter of the patients; patients supported with TandemHeart or Impella 5.0 could be bridged to long-term MCS in  >25% of cases with good long-term outcome, but only a minority of patients treated with peripheral ECMO were bridged to long-term support. According to the authors, due to heterogeneous patient populations, the use of different devices, and a lack of controlled trials, it is currently impossible to provide evidence-based recommendations on the optimal duration of temporary MCS use to bridge to durable LVAD [62].

In one study on bridge-to-bridge conversion to LVAD, patients on temporary MCS were found to have comparable end-organ function and prothrombin time, better hemodynamic profile, and improved 3-year mortality compared with the primary implant group at baseline [64]. However, the patients also had longer operative and cardiopulmonary bypass times, greater volume of perioperative blood transfusion, and longer duration of index hospitalization. The incidence of pump infection, systemic infection, and stroke were also higher in the temporary MCS group. Ultimately, while long-term MCS support increases the risk of post-bridge device-related morbidity and mortality, optimization of end-organ function and hemodynamics requires a certain period of temporary MCS support in severely ill heart failure patients. Thus, there is a need to innovate more sophisticated BTB surgical techniques that minimize operative time, infection risk, and blood transfusions. Guidelines on the timing of BTB surgery and appropriate patient selection might also improve survival. In particular, ECMO before LVAD implantation has been independently associated with poor survival [65].

High early mortality rate after bridged implantation has been previously attributed to unrecognized right ventricular failure [66]. Thus, it is possible that hemodynamic indices do not reflect true RV function, further evidence that assessment of RV function is essential before durable LVAD insertion. Additionally, non-sternotomy approaches may have a beneficial effect on post-LVAD RVF by avoiding excessive distension of the RV both during implant and by keeping pericardial restraint in place postoperatively [67,68]. These findings also highlight the importance of administering optimal heart failure medication and evaluating serial cardiac function for possible myocardial recovery after durable LVAD implantation in selected patients who initially present with refractory cardiogenic shock and acute heart failure [66]. Reducing infection at the cannulation site of the temporary support device has been emphasized as a critically important issue for minimizing postoperative infection and need for reoperation [64].

## 3. Considerations for Heart Transplantation

### 3.1. Bridge to Transplant with Temporary Mechanical Circulatory Support

The goal of temporary MCS devices is to provide adequate end-organ perfusion and unload the ventricle. For patients on temporary MCS, the benefits and risks of continued support should be weighed, and readiness to wean should be assessed regularly.

Depending on the patient’s hemodynamics and echocardiography findings, MCS support may need to be escalated or de-escalated. Intra-aortic balloon pumps are usually inserted percutaneously through the femoral artery and provide 0.5 L/min of flow [2]. Compared to other MCS devices, IABPs provide lower hemodynamic support, but advantages of this device include ease of insertion and low cost. VADs can be temporary or durable and can support the left or right ventricle. Hemodynamic support and flow volumes vary based on the type of VAD utilized, with some devices, such as the Impella 5.5 (Abiomed, Danvers, MA, USA) providing up to 6.2 L/min of flow. VA-ECMO provides biventricular support and oxygenation. Of all temporary MCS devices, VA-ECMO provides the greatest hemodynamic support with up to 10 L/min of flow.

In 2018, the United Network for Organ Sharing (UNOS) revised their heart allocation policy. Under the revised allocation policy, patients supported with temporary MCS devices were assigned a higher priority status (status 1 or 2). Following this change, the number of patients bridged with temporary MCS devices has increased. Candidates supported with IABPs are assigned status 2 and make up 27% of transplant recipients in the post-2018 policy change era. Candidates supported with temporary VADs are also assigned status 2 and now make up 4% of transplant recipients [69]. Candidates supported with VA-ECMO are assigned status 1 and make up 5.3% of all transplant recipients.

While on MCS support, patients may face a number of complications. The early experience with the Impella 5.5 device found that over a third of patients required transfusions for bleeding [70], which could result in sensitization for candidates on the waitlist. VA-ECMO is associated with bleeding, thrombosis, and high mortality on the waitlist. However, studies of post-transplant outcomes have found that those bridged to transplant with temporary MCS have excellent survival, with similar outcomes as non-bridged recipients [71]. Recipients bridged with ECMO in particular have had significant improvements in post-transplant outcomes following the 2018 policy revision [72,73].

### 3.2. Advanced Recipient Age

With an aging population, the age of heart transplant candidates and recipients has also been increasing. Recipients ≥ 70 years now make up over 10% of the heart transplant candidate population [74]. As the age of heart transplant candidates and recipients continue to increase, a number of preoperative, operative, and postoperative factors must be considered.

Pre-transplant considerations include the increased number of comorbidities, with increased rates of hypertension, prior malignancy, and chronic kidney disease in those over the age of 70 compared to younger candidates [74]. Additional pre-transplant considerations for this population include cognitive function evaluation, caregiver support, and frailty. Interestingly, older candidates are often less acutely sick at the time of transplant, with lower rates of mechanical circulatory support at the time of transplant compared to younger recipients [74]. This likely reflects a bias towards choosing older candidates who were healthier and more likely to be able to benefit from a heart transplant.

Operative considerations for older recipients include donor characteristics. Many transplant programs are more willing to utilize extended criteria donors for older recipients. Past studies have demonstrated that donors for older (vs. younger) candidates have had a higher median age (36 vs. 30 years) and were more likely to have a history of diabetes and hypertension [74].

National studies have demonstrated excellent outcomes post-transplant in recipients over the age of 70. Jaiswal et al. demonstrated worse unadjusted 5-year survival in recipients ≥ 70 years compared to younger recipients [74]. However, no difference was observed on adjusted analysis. Jaiswal et al.’s analysis mirrors other contemporary studies in finding similar long-term post-transplant survival in recipients ≥ 70 years compared to younger recipients; these studies are summarized in Table 2 [74,75,76]. Older recipients are, however, more likely to experience post-transplant stroke [74], more likely to die of infection or malignancy compared to younger recipients [77], and less likely to die of acute rejection [77]. These results are likely a reflection of the established age-related decline in immune function and highlight a potential role for age-based tailoring of immunosuppression to decrease infection and malignancy in older recipients.

### 3.3. Elevated Body Mass Index

Given the growing prevalence of obesity in the United States and its association with other comorbidities [78], the impact of increased body mass index (BMI) on heart transplant candidates carries important implications for care and access to transplant.

Pre-transplant considerations for patients with elevated BMI include comorbidities, donor–recipient size matching, and bridge-to-transplant listing strategy. The literature on heart transplant candidates with BMI 30–35 kg/m^2^ is mixed [79], but the International Society of Heart and Lung Transplantation (ISHLT) 2016 guidelines recommend weight loss for heart transplant candidates with class II obesity (BMI > 35 kg/m^2^) before listing [80]. Candidates may undergo LVAD implantation or bariatric surgery [81] to promote weight loss, although patients rarely achieve weight loss post-LVAD [82] and may experience adverse outcomes from delayed listing. Although candidates with obesity may be listed at a lower status due to higher LVAD use and lower use of IABP and mechanical/inotropic support, under the current 2018 allocation system, patients with BMI > 30 kg/m^2^ have experienced improved waitlist times, transplantation rates, and waitlist mortality [83]. The ISHLT also recommends that the donor body weight be no more than 30% (male) or 20% (female) below that of the recipient [84], which reduces the availability of potential hearts to a candidate with elevated BMI.

Intraoperatively, heart transplant recipients with BMI > 35 kg/m^2^ are associated with increased cardiopulmonary bypass times [79], which has been associated with higher ICU length of stay and in-hospital mortality in other cardiac surgery patients [85]. In heart transplant recipients, a BMI > 30 kg/m^2^ has been associated with longer waitlist times and increased risk of all-cause mortality post-transplant, with comorbidities such as diabetes, dyslipidemia, and hypertension adversely impacting outcomes [86]. Although the literature on specific post-transplant complications in heart transplant recipients with obesity is limited, Nagendran et al. found that heart transplant recipients with a BMI > 35 kg/m^2^ are more likely to develop postoperative infection and early complications in general (<30 days post-heart transplant) [79].

### 3.4. Multiorgan Transplantation

The frequency of multiorgan transplantation in heart transplant has been steadily increasing since the 1990s. In 2023, multiorgan heart transplants comprised 11% of all heart transplants (504 of 4545), with the majority (84%) being combined heart–kidney transplants [87].

Preoperative considerations for multiorgan heart transplant include the degree of second organ dysfunction, current and historical trends of second organ function, comorbidities associated with irreversible second organ damage (e.g., diabetes and lupus for kidney injury), and center volume [88,89]. While the individual benefit to multiorgan heart transplantation is well documented in the literature, equitable organ allocation should be considered by assessing whether the second organ dysfunction will improve once cardiac function is restored through heart-only transplantation. For heart–kidney transplant, this evaluation is based on the glomerular filtration rate and evidence of chronic kidney disease; for heart–liver, factors include liver function tests, imaging, and biopsy [88]. To address concerns about equitable organ allocation in multiorgan heart transplantation, UNOS implemented a new allocation policy for heart–kidney and heart–lung in June 2023 that adds priority to recipients who are listed for kidney or lung transplants after their heart transplant with the goal of reducing unnecessary simultaneous heart–kidney transplantation and allocating organs more equitably [90].

Operative considerations for multiorgan heart transplant include anatomic planning and the timing of the second organ transplant. In heart–kidney transplants, anatomic planning includes preoperative imaging to determine arterial targets for implanting the kidney allograft and keeping one groin free of vascular catheters for allograft placement. For the timing of the second organ transplant, kidney transplantation may be immediate or staged after heart transplant, while liver transplantation most commonly occurs sequentially after the patient is weaned from cardiopulmonary bypass after heart transplant, although en bloc heart–liver transplantation also occurs with comparable outcomes [88].

Although postoperative care for multiorgan heart transplant may be more complex, as evidenced by extended hospital lengths of stay for these patients, overall post-transplant outcomes including overall survival, cardiac allograft vasculopathy, and graft dysfunction have been more favorable compared to heart-only transplantation [91,92]. Heart–kidney transplantation is also a favorable strategy for retransplantation, comprising 12.8% of retransplants compared to 2.4% heart-only retransplants in 2016 [91]. The immunosuppressive strategy for multiorgan transplantation varies by organ; for heart–kidney, it involves induction immunosuppression using calcineurin inhibitors (primarily tacrolimus to reduce nephrotoxicity), while for heart–liver, induction immunosuppression is not indicated [88]. Multiorgan heart recipients should be carefully monitored for signs of infection after transplant, as an enhanced level of immunosuppression may increase their susceptibility to infection, comprising the primary cause of death among multiorgan recipients [91].

### 3.5. Prior Cardiac Surgery

Research on heart transplantation in patients with prior cardiac surgery remains sparse. In one study of 61 heart transplant recipients, those who had previous nontransplant cardiac operations were found to require a significantly longer cardiopulmonary bypass time and operative time [93]. Additionally, patients undergoing a second transplantation were identified as a high-risk subset with higher operative mortality and lower 1-year survival [93]. Other studies comparing redo-sternotomy heart transplant recipients versus patients without prior cardiac surgery have reported increased intraoperative blood utilization, postoperative blood loss, and hospital length of stay [94,95], as well as decreased early, mid, and long-term survival [95,96]. Published outcomes from studies examining reoperative heart transplant are summarized in Table 3. The longer bypass duration, need for intraoperative blood products, and perioperative mortality reported in these patients reflects the operative complexity of heart transplantation in these patients. Reoperative surgery is associated with dense adhesions and distorted anatomy, leading to a potentially increased risk of injury to the heart and great vessels and may lead to the decision to pursue alternate methods of cannulation [95]. Care should be taken in the operative approach to transplantation in these patients, and these patients should be monitored closely for bleeding in the early postoperative period.

Bridge-to-transplant left ventricular assist device therapy prior to heart transplantation has yielded positive outcomes but also requires redo-sternotomy. LVAD therapy can provide effective hemodynamic support for patients awaiting heart transplant despite a significant potential for adverse events [2,97]. One study that compared heart transplant outcomes for patients bridged to transplantation with an LVAD to primary heart transplantation patients found exceptionally high survival in LVAD patients [98], though other studies have suggested a negative influence of prolonged LVAD support on post-transplant outcomes [99,100]. The most common causes of death which contribute to early mortality in these patients have been observed to be cardiovascular death, infection, and multisystem organ failure within the first year after transplant [100]. In a study of 54 LVAD patients who underwent heart transplant, antimicrobial therapy was extended to treat preceding infections in nine cases of LVAD-specific infection for a median duration of 14 days [101]. Although infection rates have decreased with recent developments in LVAD technology, aggressive strategies for prevention and treatment of infection must continue to be refined. As antibiotic-resistant organisms are frequently the source of these device-related infections, preventative measures are likely to have a significant impact on infection rates [102].

The optimal timing of heart transplantation following LVAD therapy has also been a recent topic of interest. One study of 468 LVAD heart transplant patients found no significant difference in survival based on LVAD duration, however patients requiring more than two units of packed red blood cells in 24 h during LVAD support had a statistically significant decreased 1-year survival [103]. Additionally, it has been demonstrated that transplanting a patient before recovery and <30 days since LVAD implant will lead to poor outcomes [104]. Moreover, since the likelihood of adverse events increases with the duration of LVAD support, transplanting before 9 to 12 months (and as early as 1–3 months) post-LVAD could also help limit complications and improve post-transplant outcomes [104]. Ultimately, in patients with advanced heart failure, the benefits of improved survival from LVAD while awaiting heart transplantation must be weighed against the risks of post-transplant mortality in heart transplant candidates. Patient bridged with mechanical support may require more careful consideration for transplant eligibility after LVAD placement.

### 3.6. Adult Congenital Heart Disease

Heart transplant in adults with congenital heart disease (ACHD) is complicated by operative complexity and a lack of center and surgeon experience. ACHD patients may have a wide range of pathology, and transplant may be challenging for these patients due to varied anatomy and operative history [105]. In a meta-analysis, Doumouras et al. found that ACHD patients, and Glenn and Fontan patients in particular, to be at increased risk of 30-day mortality compared to non-ACHD patients [106]. Univentricular ACHD patients without Glenn or Fontan procedures had similar post-transplant survival to non-ACHD patients, suggesting that early mortality after transplant could be attributed to these patients’ palliative surgical history, which may necessitate additional pulmonary artery, systemic venous, and/or great artery reconstruction at the time of transplant [105]. In Fontan patients, transplant must be weighed carefully with Fontan revision in patients experiencing ventricular dysfunction, and there is often uncertainty as to the degree of ventricular dysfunction that precludes Fontan revision [105]. Systolic function is difficult to measure in these patients due to indeterminate ventricular morphology. Additionally, when evaluating ACHD patients for transplant, patients with high exposures to blood products in prior operations and those with implanted allograft tissue during prior operations are at increased risk for rejection due to high levels of circulating antibodies. ACHD patients requiring transplant should be evaluated at experienced transplant centers due to the complex decision-making surrounding their operations and the established impact of center volume on their outcomes [107].

### 3.7. Increased Risk Donors

As with all solid organ transplantation, heart transplantation continues to be limited by a shortage of donor organs. In recent years, efforts to expand the donor pool have been made through the utilization of increased risk donors. For heart transplantation, these have included donation after circulatory death (DCD) donors, hepatitis C (HCV), and donors with increased ischemic times.

DCD donors have remerged in cardiac transplantation over the last three years as organ procurement and perfusion techniques have improved. DCD donors can be procured using direct procurement and perfusion, which involves ex situ perfusion of the heart using a machine, or using normothermic regional perfusion, which involves an in situ perfusion interval using ECMO or cardiopulmonary bypass. Results from a recent randomized noninferiority trial have demonstrated that the use of DCD donors for heart transplantation results in a similar risk-adjusted 6-month survival compared to brain death donors [108].

With the approval of direct acting antivirals (DAAs) in the last decade, the utilization of HCV+ donors has become more widespread. For those receiving HCV NAT+ donors, the transmission rate of HCV for heart transplant recipients has been shown to be 95.7% [109] with a median of 5 days between transplant and detection of viremia. Treatment is typically initiated post-transplant, with a median time from transplant to initiation of treatment of about 55 days [109]. Common DAA regimens for the treatment of donor-derived HCV in this population include ledipasvir–sofosbuvir (90–400 mg), sofosbuvir–velpatasvir (400–100 mg), and glecaprevir–pibrentasvir (100–40 mg) with a duration of 12 weeks. Single-center studies have demonstrated that recipients who seroconvert following transplant are successfully treated with DAA regimens [109]. Other national studies have consistently demonstrated excellent post-transplant survival up to 3 years [110,111].

Additional studies have investigated increased ischemic times above 4 h. A study by Yeen et al. demonstrated similar survival between transplants with ischemic times less than 4 h and those with ischemic times between 4 and 5 h [112]. However, those with ischemic times of greater than 5 h were found to have decreased survival. Prolonged ischemic times have been shown to have a greater impact on older recipients, recipients on ECMO, recipients on dialysis, or recipients with ischemic etiology. New controlled hypothermic preservation methods have additionally been developed, with results showing significant reduction in the risk of primary graft dysfunction at all ischemic times compared to static cold storage [113]. Controlled hypothermic preservation may allow for the recovery of hearts from distant locations with less concern about the negative impact of prolonged ischemic times [114].

## 4. Conclusions

For patients with end-stage heart failure, definitive therapies include durable LVADs and heart transplantation. For durable LVADs, a number of important considerations must be made for special populations, including patients with RV failure, history of stroke, small LV cavity, preoperative arrhythmias, pre-existing valvular disease, and temporary MCS bridging. For those undergoing heart transplantation, attention must be paid for those bridged with temporary MCS, advanced age (≥70 years) recipients, multiorgan transplant recipients, those with prior cardiac surgery, ACHD recipients, and those with elevated BMI. There are also special concerns regarding the use of increased risk donors, such as DCD donors, HCV positive donors, and donors with increased ischemic time. As both heart transplantation and the use of durable LVADs as destination therapy continue to increase, it will be important to take into account these considerations in clinical practice and continue to assess improvements in care for these special populations.

## Figures and Tables

**Table 1:** Published risk prediction models for right ventricular failure after LVAD implantation.

Study	Study Population	Discrimination (C-Statistic)	Rate of RVF	Score Components
EuroMACS-RHF risk score (2017)	*n* = 2000 100% continuous-flow 17% destination therapy	0.70	21.7%	Hemoglobin ≤ 10 Ratio of right atrial to pulmonary capillary wedge pressure > 0.54 Severe RV dysfunction on echocardiography INTERMACS class 1–3 Use of multiple inotropes
Pittsburgh decision tree (2012)	*n* = 183 21.9% continuous-flow % destination therapy not reported	0.87	15%	Number of inotropic agents Alanine aminotransferase White blood cell count Heart rate International normalized ratio Right atrial pressure Age Transpulmonary gradient
Utah RVF risk score (2010)	*n* = 175 14% continuous-flow 42% destination therapy	0.74	44%	Beta blocker ACE inhibitor/angiotensin receptor blocker Obesity Inotrope dependency Pulmonary vascular resistance IABP Destination therapy
Michigan RVF risk score (2008)	*n* = 197 14% continuous-flow 6% destination therapy	0.73	35%	Creatinine ≥ 2.3 mg/dL Bilirubin ≥ 2.0 mg/dL Aspartate aminotransferase ≥ 80 IU/L Vasopressor requirement

**Table 2:** Published heart transplant outcomes in recipients aged ≥70 years.

Study	1 Year Survival	5-Year Survival
Jaiswal et al. (2021) [74] *n* = 57,285 [806 ≥70 years vs. 36,329 <70 years]	88.5% [≥70 years] vs. 89.6% [<70 years], *p* = 0.13	79.6% [≥70 years] vs. 80.8% [<70 years] aHR for mortality 1.06 [95% CI: 0.91–1.25], *p* = 0.43
Cooper et al. (2017) [75] *n* = 50,432 (715 ≥70 years vs. 13,527 60–69 years vs. 36,190 18–59 years]	--	69.2% [≥70 years] vs. 70.7% [60–69 years] vs. 73.1% [18–59 years] aHR for mortality (≥70 years vs. 60–69 years) 1.13 [95% CI: 0.90–1.41], *p* = 0.28 aHR for mortality (≥70 years vs. 18–59 years) 1.24 [95% CI: 1.00–1.54], *p* = 0.05
Daneshvar et al. (2011) [76] *n* = 519 [37 ≥70 years vs. 206 60–69 years vs. 276 ≤60 years]	94.6% [≥70 years] vs. 92.7% [60–69 years] vs. 92.0% [≤60 years], *p* = 0.25	83.2% [≥70 years] vs. 73.8% [60–69 years] vs. 74.7% [≤60 years], *p* = 0.25

Abbreviations: aHR, adjusted hazard ratio.

**Table 3:** Published impact of prior cardiac surgery on heart transplant outcomes.

Study	CPB Time (Minutes)	Re-Exploration for Bleeding	Perioperative Blood Products	Operative Mortality	1-Year Mortality	5-Year Mortality
Ott et al. (1994), *n* = 155 [61 reoperative vs. 85 first-time] [93]	**128 vs. 45, *p* < 0.01**	0.0% vs. 0.01%	51.7% vs. 44.0% required > 2 units, *p* = 0.36	6.6% vs. 4.7%, *p* > 0.9	85.3% vs. 87.1%, *p* > 0.9	76.0% vs. 72.9%, *p* > 0.9
Ott et al. (1994), *n* = 94 [9 re-transplant vs. 85 first-time] [93]	**--**	**--**	**--**	**22.8% vs. 4.7%, *p* < 0.001**	**33.3% vs. 87.1%, *p* < 0.001**	--
Aziz et al. (2000), *n* = 156 [49 reoperative vs. 107 first-time] [94]	**134 vs. 82, *p* = 0.02**	**20% vs. 4%, *p* = 0.004**	4.5 vs. 3.6 units, *p* = 0.3	12.5% vs. 13%, *p* = 0.9	83% vs. 83%, *p* = 0.9	68% vs. 71%, *p* = 0.9
George et al. (2012), *n* = 631 [356 reoperative vs. 275 first-time] [95]	**191 vs. 156, *p* < 0.001**	--	**26.5 vs. 18 units, *p* = 0.003**	**90.2% vs. 98.5%, *p* < 0.001**	**79.6% vs. 93.1%, *p* < 0.001**	**70.1% vs. 80.4%, *p* < 0.001**
Axtell et al. (2019), *n* = 14,730 [7365 reoperative vs. 7365 first-time] [96]	**--**	--	--	--		HR for mortality **1.13 (95% CI: 1.05–1.22), *p* < 0.001**

Significant values are bolded. Abbreviations: CPB, cardiopulmonary bypass; HR, hazard ratio.
